# Multi-Parameter Estimation Method and Closed-Form Solution Study for *k-µ* Channel Model

**DOI:** 10.3390/s23104760

**Published:** 2023-05-15

**Authors:** Jie Tian, Zhongqing Fan, Zhengyu Ji, Xianglu Li, Peng Fei, Dong Hou

**Affiliations:** 1 Institute of Electronic Engineering, China Academy of Engineering Physics, Mianyang 621999, China; 2 High-Tech Institute, The First School, Rocket Force University of Engineering, Xi’an 710025, China; 3 The School of Automation Engineering, University of Electronic Science and Technology of China, Chengdu 611731, China

**Keywords:** *k-µ* distribution, fading channel model, multi-parameter estimation, closed-form solution

## Abstract

This paper proposes a novel multi-parameter estimation algorithm for the *k-µ* fading channel model to analyze wireless transmission performance in complex time-varying and non-line-of-sight communication scenarios involving moving targets. The proposed estimator offers a mathematically tractable theoretical framework for the application of the *k-µ* fading channel model in realistic scenarios. Specifically, the algorithm obtains expressions for the moment-generating function of the *k-µ* fading distribution and eliminates the gamma function using the even-order moment value comparison method. It then obtains two sets of solution models for the moment-generating function at different orders, which enable the estimation of the *k* and *µ* parameters using three sets of closed-form solutions. The *k* and *µ* parameters are estimated based on received channel data samples generated using the Monte Carlo method to restore the distribution envelope of the received signal. Simulation results show strong agreement between theoretical and estimated values for the closed-form estimated solutions. Additionally, the differences in complexity, accuracy exhibited under different parameter settings, and robustness under decreasing SNR may make the estimators suitable for different practical application scenarios.

## 1. Introduction

In wireless communication systems, the quality of the channel plays a significant role in determining the overall communication quality. The channel quality indicator (CQI) is a critical parameter that determines the maximum achievable data rate of the system [1,2]. Advances in wireless communication technology have been focused on improving channel estimation performance and enhancing modulation schemes. Currently, 5G networks are still under active development and construction. To meet the high-performance requirements of 5G communication, such as achieving Gbps-level high-speed transmission, low end-to-end transmission latency of 1 ms, and high-bandwidth instant burst transmission, the communication system requires measuring CQI and analyzing it based on the channel state information. This analysis provides theoretical support for selecting system parameters such as modulation scheme, data block size, coding method, and data rate, to optimize the system’s throughput, latency, and bandwidth performance.

Accurately reflecting changes in the physical quantity of the channel in real time is a crucial aspect of the channel quality indicator (CQI). Commonly used metrics for assessing channel quality include the bit error rate (BER) and signal-to-interference-plus-noise ratio (SINR) [3], etc. These metrics are quantified factors calculated by the user through signal reception and processing, and their accuracy depends on the quality of the channel estimation. Since signal energy propagation in wireless channels is a stochastic process, channel characteristics are typically described using probability distribution models that can adapt to measured data in real-world environments. To model the channel, the usual approach is to estimate specific model parameters from the received measurement data and then evaluate the fit between the data and the model to determine the channel characteristics. Furthermore, the time-varying nature of channels in mobile communication necessitates that the channel estimation process controls computational delay to effectively reduce the real-time impact of measurement cycles on channel evaluation. Therefore, determining the probability distribution model depends not only on the sample size but also on the parameters to be estimated, the selected distribution, and the parameter estimation method.

However, in indoor communication, the highly dynamic and complex nature of channels makes it difficult to accurately analyze channel characteristics. This is mainly due to the rich transmission multipath and random shadowing effects caused by complex environments such as indoor and cabin spaces, which result in a large number of multipath propagation components in wireless signals between communication transceivers, and the LOS path cannot be guaranteed to exist. As a result, users have difficulty determining the typical channel types of signal fading and distribution changes in different positions while moving. This leads to a decrease in the performance of channel estimators and the problem of mismatch between the estimated and measured values of the channel estimation curve, which can seriously affect system performance prediction, adaptive modulation strategy generation, and transmission capacity optimization. Therefore, a more generalized representation of channel models is needed to address these challenges.

As research has progressed, new channel models have been proposed, including the *k-µ* distribution and *k-µ* fading distribution models, which are more generalized and versatile models for channel fading. The *k-µ* distribution model, in particular, has garnered attention due to its good agreement with measured channel data and its ability to degrade to different typical channel fading models (e.g., Rayleigh, Rice, and Nakagami-m channels) under certain parameter constraints [4], the development of typical fading channel models can be seen in Table 1. Since the estimation of channel models plays a crucial role in predicting the performance of 5G communication systems, it is important to study the *k-µ* fading channel model and develop reliable and effective algorithms for estimating its parameters, which can provide a new approach for modeling channels. This is essential to analyzing the theoretical performance limits of communication systems operating in *k-µ* fading channels and providing a solid theoretical basis for system design.

In recent years, there have been many studies on channel performance analysis and system performance optimization of typical fading channel models both domestically and internationally, as reported in [1,11,12,13]. However, there have been relatively few studies on parameter estimation of the *k-µ* distribution and *k-µ* shadowing distribution models [14,15,16,17]. In [14], the authors analyzed the error vector magnitude (EVM) performance of the *k-µ* fading channel, where the EVM parameter was used to evaluate the variation of channel quality, and the results could be used to predict the lower bound of channel quality based on *k-µ* fading channels. In [15], the authors assumed QAM modulation channels passing through *k-µ* shadow fading channels and proposed the average symbol error rate (ASER) expressions for orthogonal amplitude modulation (RQAM) and cross-orthogonal amplitude modulation (XQAM) in single-input single-output (SISO) and multiple-input multiple-output (MIMO) channel systems. In [16], the transmission characteristics of different receivers in *k-µ* shadowing fading channels were studied. The average bit error rate, outage probability, and channel capacity under three different receivers, namely, N-branch selection combining, dual-branch selection combining, and dual-branch maximal ratio combining, were derived. In [17], the authors used the *k-µ* fading channel model to describe complex data transmission scenarios in a bidirectional relay network, derived the outage probability under *k-µ* fading channels, and also derived the outage probability under various mixed fading channels.

In addition, the *k-μ* distribution and *k-μ* shadowing distribution have been widely used in the performance analysis of wireless communication and network systems. Through detailed analysis of the higher-order statistical characteristics of the frequency-agile system in the *k-μ* and *k-μ* shadow fading channels, reference [18] derived novel and accurate expressions for the high-order cumulants and moments of channel capacity under the generalized fading model, as well as simplified high-order statistical expressions for channel capacity in extremely low and high signal-to-noise ratio conditions. Reference [19] considered a master-slave and cognitive relay spectrum sharing network in which the channel follows a *k-μ* shadow fading distribution, derived the expression for the interruption probability of the cognitive relay network under the peak interference power constraint of the master user network, and analyzed the system performance of the cognitive relay network under different fading and shadowing parameters and power constraint conditions. Reference [20] studied the performance of energy-based spectrum sensing in *k-μ* and *k-μ* extreme fading channels under the Bayesian framework, and derived accurate expressions for the channel model error probability (as a convex combination of false alarm probability and signal detection probability). Reference [21] derived a novel closed-form analytical solution for the average quality metric (average receiver operating characteristic curve and area under the curve) of a generic *k-μ* shadow fading channel model by analyzing the average quality metric describing the energy detection process; moreover, to investigate the impact of human body occlusion on the channel in cellular device-to-device communication, reference [22] derived the probability density function and moment function of the channel model and applied it to link communication between devices in an outdoor urban environment at 868 MHz, and found that the shadow effect of the deterministic component varies significantly depending on the relative position of the user device to the body and link geometry. Reference [23] derived approximate expressions for the system interruption probability and channel capacity in a system where both the sensing transmission signal and the interference signal experience *k-μ* shadow fading under given conditions such as signal-to-noise ratio. These expressions are expressed in known hypergeometric functions and can be easily calculated numerically. However, while most of these studies evaluate channel transmission quality in specific scenarios through the analysis of system interruption probability, average bit error rate, and channel capacity based on *k-μ* distribution and *k-μ* shadow distribution, they do not provide effective channel model parameter estimation methods by assuming the range of critical channel parameters such as *k* and *µ*, making it difficult to provide adjustment strategies for communication system transmission scheme design and performance analysis optimization through accurate channel estimation and CQI quantization in practical scenarios, thus affecting system generality.

In the existing research on *k-μ* parameter estimators, Yacoub et al. [24] proposed closed-form expressions for *k-μ* parameters, and Ribeiro et al. further analyzed the asymptotic validity of this estimator [25]. However, our experiments have shown that this estimator is inefficient and prone to errors in estimation, which means the method in [24] has a higher probability of error when estimating channel parameters. [25] includes transcendental functions that incorporate special functions such as gamma and hypergeometric functions, which means that they cannot be solved analytically, but only through numerical methods. Additionally, a maximum likelihood-based α-η-μ distribution parameter estimator was proposed in [26] for inferring the characteristics of signal amplitude distribution. However, this study’s conclusion is not a closed-form expression, and the algorithm’s complexity is high. To address these issues, this paper proposes a new *k-μ* parameter estimator for the communication system’s *k-µ* fading channel model, providing theoretical support for distinguishing fading channel types. The main contributions of this paper are as follows: First, two mathematical models containing the unknown parameters *k* and *µ* are established using the moment-generating function. Second, three *k-μ* parameter moment estimators based on the *k-µ* fading model are derived, resulting in corresponding closed-form expressions for parameter estimation. The complexity and estimation accuracy of the estimator are analyzed. Finally, simulation analysis is performed on different parameters, ensuring high-precision and effective estimation of *k* and *µ* parameters under different *k*-value conditions.

The structure of this paper is organized as follows: In Section 2, we introduce the *k-μ* distribution-based fading channel model and the existing estimators. In Section 3, we provide the derivation process for three moment-based estimators. In Section 4, we utilize Monte Carlo simulation to generate test data and compare the computational complexity and estimation accuracy of the three moment-based estimators proposed in this paper with the estimator proposed by Yacoub. We also discuss the applicability of each estimator in different scenarios.

## 2. *k-µ* Fading Channel Models

In wireless communication systems, the received signal at the receiver can be represented as [27]

(1) y(t)=x(t)⊗h(t)+n(t)

where x(t) is the transmit signal, h(t) is the channel impulse response (CIR) signal, and n(t) is the noise. The CIR signal describes the energy attenuation and time delay of countless electromagnetic waves that undergo optical phenomena such as reflection and diffraction in the channel, and then arrive at the receiver. Energy attenuation and time delay are two uncorrelated random variables that follow different probability distributions. Various channel fading models (such as Rice, *Nakagami*-*M*, etc.) use statistical methods to describe the energy attenuation random variable. Assuming that a known frequency-domain signal X(f) is transmitted from the transmitter and undergoes multipath fading, the received signal filtered at the receiver can be represented in the frequency domain as Y(f)=X(f)⋅H(f)+N(f). The estimated channel state information (CSI) obtained from it is $\hat{H}(f)=X^{-1}(f)\cdot [Y(f)-N(f)]$, which is the channel frequency response (CFR). By performing the inverse Fourier transform on $\hat{H}(f)$, the channel impulse response (CIR) can be obtained and denoted as $\hat{h}(t)$, and r=|h(t)|.

The classical Rician channel model assumes that the obstacles in the channel follow a uniform distribution, and it defines the signal that arrives at the receiver as a cluster; there is no time correlation between each electromagnetic wave, and the energy fading of the electromagnetic wave obeys a normal distribution and the phase follows a uniform distribution. However, under practical scenario conditions, the electromagnetic waves that arrive at the receiver will be time-dependent because of the non-uniform distribution of the obstacles in the channel, and the CIR will show multiple energy peaks in the experimental data. According to the multiple-cluster effects of the transmitted electromagnetic waves arriving at the receiving end, the electromagnetic waves can be divided into a set of clusters; the number of clusters is denoted by *μ*, and each cluster is individually subject to the Rice distribution. This new method for modeling electromagnetic waves was first proposed by Yacoub [10] and is called the *k*-*µ* fading distribution model. The cumulative electrical frequency amplitude (envelope) of the *µ* clusters of electromagnetic waves that arrive at the receiver satisfies the following physical model [24].

(2) $$r^{2}=\sum_{i=1}^{\mu }{\left(p_{i}+x_{i}\right)}^{2}+\sum_{i=1}^{\mu }{\left(q_{i}+y_{i}\right)}^{2}$$

where $r^{2}$ is the instantaneous power of the carrier signal at the receiving end, *μ* is the number of clusters of electromagnetic waves. *p_i_*, *q_i_* are the mean values of the homogeneous and quadrature components of the *i*-th cluster of multipath waves, respectively, and $p_{i}{}^{2}$, $q_{i}{}^{2}$ are the real and imaginary parts of the *i*-th cluster of the deterministic component signal. *x_i_*, *y_i_* are the real and imaginary parts of the scattered wave signal in the *i*-th cluster signal, respectively, which are the mutually independent Gaussian processes and satisfy the relationship of $E[x_{i}]=E[y_{i}]=0$ and $E[x_{i}^{2}]=E[y_{i}^{2}]=\sigma^{2}$. Therefore, the probability distribution function (PDF) of the *k*-*µ* distribution can be expressed as [24]

(3) $$f(\alpha )=\frac{2\mu {\left(1+k\right)}^{(\mu +1)/2}\cdot \alpha^{\mu }}{k^{(\mu -1)/2}e^{\left(\mu k+\mu (1+k)\alpha^{2}\right)}}I_{\mu -1}(2\mu \sqrt{k(1+k)}\alpha )$$

where α is the normalized envelope and satisfies the relationship of $\alpha =r/\sqrt{E[r^{2}]}$. According to the probability distribution conversion relationship in probability theory, the PDF of the *k*-*µ* fading distribution with respect to the envelope *r* can be expressed as:

(4) $$f(r)=\frac{f(\alpha )}{\sqrt{\Omega }}=\frac{2\mu {\left(1+k\right)}^{(\mu +1)/2}\cdot r^{\mu }}{{\sqrt{\Omega }}^{\mu +1}\cdot k^{(\mu -1)/2}e^{\left(\mu k+{\frac{\mu (1+k)r}{\Omega }}^{2}\right)}}I_{\mu -1}(2\mu \sqrt{k(1+k)}\frac{r}{\sqrt{\Omega }})$$

the parameter Ω represents the average signal power $\Omega =E[r^{2}]$. When the number of clusters *µ* is a natural number, *k* represents the ratio of the deterministic component to the non-line-of-sight path component (or scatter component). When *k* = 0 and *µ* = *M*, the *k-µ* fading distribution degenerates to the Nakagami-M distribution. When *k* = *K* and *µ* = 1, the *k-µ* fading distribution becomes the Rician distribution. When *k* = 0 and *µ* = 1, the *k-µ* fading distribution becomes the Rayleigh distribution. Applying $E[r^{n}]={\bar{r}}^{n}\cdot E[\alpha^{n}]={\sqrt{E[r^{2}]}}^{n}\cdot E[\alpha^{n}]$ from ([19], Equation (6)) to the function from ([20], Equation (5)), the moment-generating function (MGF) for the *k-µ* distribution can be expressed as:

(5) E[rn]=E(r2)n⋅Γ(μ+n/2)⋅exp(−kμ)Γ(μ)⋅[(1+k)μ]n/2F11(μ+n/2,μ;kμ)

Here, *n* denotes the order of the moment function, F11( · , · ; ·) represents the Kummer confluent hypergeometric function, and Γ(⋅) represents the gamma function. In previous research, Yacoub has proposed a closed-form estimator for the parameter *k* in ([24], Equation (12)), and demonstrated the mapping relationship between *k* and *μ* in ([24], Equation (9)), as shown below:

(6) $${\hat{k}}_{yacoub}={\left(\frac{\sqrt{2}(E[\alpha^{4}]-1)}{\sqrt{2E{\left[\alpha^{4}\right]}^{2}-E[\alpha^{4}]-E[\alpha^{6}]}}-2\right)}^{-1}$$

(7) $${\hat{\mu }}_{yacoub}=\frac{1}{Var[\alpha^{2}]}\frac{1+2{\hat{k}}_{yacoub}}{{\left(1+{\hat{k}}_{yacoub}\right)}^{2}}$$

In the above equation, *Var*[·] denotes the variance operator, and $Var[\alpha^{2}]=$ $Var[r^{2}]/E^{2}[r^{2}]$. Based on the equation given in reference $E[r^{n}]={\bar{r}}^{n}\cdot E[\alpha^{n}]$ $={\sqrt{E[r^{2}]}}^{n}\cdot E[\alpha^{n}]$ ([19], Equation (6)), when the total signal power is $E[r^{2}]=1$, the variable α in Equations (6) and (7) can be exchanged with the envelope r.

## 3. The Derivation and Design of the *k*-*μ* Estimators

The moment estimation method has always been one of the most commonly used methods for extracting channel parameters, owing to its advantages of closed-form simplicity, low algorithm complexity, and small estimation error. It has been widely used in the research of fading channel model parameter estimators, such as the *K*-factor estimator based on the Rice distribution [28], the M-parameter estimator based on the Nakagami distribution [29], and the *k*-parameter estimator based on the *k-µ* distribution [25], among others. Moreover, the various-order moment values of the signal envelope can be easily obtained through experimental data. As the sample size approaches infinity, the theoretical *n*-th moment value and the expected value of the *n*-th power of the measured data have unbiased asymptotic properties. Therefore, in this paper, we choose the moment estimation method to estimate the *k* and *µ* parameters of the *k-µ* distribution.

From the moment functions of the *k-μ* distribution in Equation (5), it is evident that each order of moment functions contains gamma functions and Kummer confluent hypergeometric functions. The expansion of the right-hand side of the moment function equation is a complex and irreducible combination polynomial, making it difficult to solve for *k* and *μ* using inverse function methods or substitution-based elimination techniques. At this point, we observe that even-order moments share the same functional structure. By utilizing two even-order moment functions as the numerator and denominator, we can eliminate the gamma function terms and exp(-*kμ*) terms in Equation (5), thus obtaining the simplified second-, fourth-, and sixth-order moments of the kappa-mu distribution (for the detailed derivation process, please refer to Appendix A).

(8) $$E(r^{2})=\Omega$$

(9) $$E(r^{4})=\Omega^{2}\cdot \left(1+\frac{1+2k}{\mu {\left(1+k\right)}^{2}}\right)$$

(10) $$E(r^{6})=\Omega^{3}\cdot \left(\frac{\mu (\mu +1)(\mu +2)+3k\mu (\mu +1)(\mu +2)+3{\left(k\mu \right)}^{2}(\mu +2)+{\left(k\mu \right)}^{3}}{{\left(1+k\right)}^{3}\mu^{3}}\right)$$

Equations (8)–(10) form a channel parameter estimation model with respect to variables *k* and *μ*. In this model, Ω represents the total signal power, while $E(r^{2}),E(r^{4}),E(r^{6})$ can be estimated from either empirical measurements or simulation data. Transforming Equation (9) into a function with respect to *μ*, we obtain:

(11) $$\mu =\frac{E^{2}(r^{2})}{Var[r^{2}]}\frac{{\left(1+k\right)}^{2}}{\left(1+2k\right)}=\eta \frac{{\left(1+k\right)}^{2}}{\left(1+2k\right)}$$

where $\eta =E^{2}(r^{2})/Var[r^{2}]$, *Var*[·] denotes the variance operator. By substituting Equation (11) into the sixth-order moment function given by Equation (10), we can simplify it to obtain Equation (12) as:

(12) $$\frac{E(r^{6})}{E^{3}(r^{2})}=\frac{\left(2+6k)(1+k\right)}{\eta^{2}{\left(1+2k\right)}^{2}}+\frac{3}{\eta }+1$$

Expanding Equation (12) allows us to obtain that

(13) $$[4(\frac{E(r^{6})}{E^{3}(r^{2})}\eta^{2}-3\eta -\eta^{2})-6]\cdot k^{2}+[4(\frac{E(r^{6})}{E^{3}(r^{2})}\eta^{2}-3\eta -\eta^{2})-8]\cdot k+(\frac{E(r^{6})}{E^{3}(r^{2})}\eta^{2}-3\eta -\eta^{2})-2=0,$$

Considering the initial conditions that *k* and *μ* must both be greater than zero, we can eliminate an invalid solution concerning *k*. Consequently, our first closed-form estimate for *k* and *μ* derived in this paper is as follows, denoted by a delta subscript (Δ):

(14) $$\left\{\begin{array}{l} {\hat{k}}_{\Delta }=\frac{\Delta +\sqrt{\Delta }}{2-2\Delta }\\ {\hat{\mu }}_{\Delta }=\eta \frac{\left(1+2{\hat{k}}_{\Delta }\right)}{{\left(1+{\hat{k}}_{\Delta }\right)}^{2}} \end{array}\right.,\left\{\begin{array}{l} \eta =\frac{E^{2}(r^{2})}{Var(r^{2})}\\ \Delta =4-2(\frac{E(r^{6})}{{\left[E(r^{2})\right]}^{3}}\eta^{2}-3\eta -\eta^{2}) \end{array}\right.,$$

Simultaneously, we define the received power $E(r^{2})=P_{1}+P_{2}$ in the *k-µ* distribution, where *P*_1_ represents the energy of the line of sight (LOS) path and *P*_2_ corresponds to the energy of the non-line-of-sight (NLOS) paths. Parameter $k=\sum_{1}^{n}\left(p_{i}^{2}+q_{i}^{2}\right)/\sum_{1}^{n}\left(x_{i}^{2}+y_{i}^{2}\right)=P_{1}/P_{2}$, the second-, fourth-, and sixth-order moments can be further transformed as follows (for a detailed derivation process, please refer to Appendix B):

(15) $$E(r^{2})=U_{2}=P_{1}+P_{2}$$

(16) $$E(r^{4})=U_{4}=\frac{\mu +1}{\mu }U_{2}{}^{2}-\frac{1}{\mu }P_{1}^{2}$$

(17) $$E(r^{6})=U_{6}=\frac{\left(\mu +1)(\mu +2\right)}{\mu^{2}}U_{2}{}^{3}-\frac{3(\mu +2)}{\mu^{2}}P_{1}^{2}U_{2}+\frac{4}{\mu^{2}}P_{1}^{3}$$

Equations (15)–(17) constitute a solution model with respect to the LOS path energy *P*_1_ and *μ*. And *U*_2_, *U*_4_, and *U*_6_ can be obtained from either measurement data or simulation data.

Let $E[r^{2}]=U_{2},E[r^{4}]=U_{4},E[r^{6}]=U_{6}$, and transform Equation (16) into the following form of function:

(18) $$\mu =\frac{U_{2}^{2}-P_{1}^{2}}{U_{4}-U_{2}^{2}}$$

Substituting this function into Equation (17) allows us to eliminate *μ*, resulting in an equation concerning *P*_1_:

(19) $$\begin{array}{ll} 0= & [U_{2}{}^{3}-3U_{2}(U_{2}{}^{2}-U_{4})-U_{6}]\cdot P_{1}^{4}+4[U_{4}{}^{2}-2U_{4}U_{2}{}^{2}+U_{2}{}^{4}]\cdot P_{1}^{3}\\ & +[U_{2}{}^{3}(U_{2}{}^{2}-3U_{4})-3U_{2}(2U_{4}{}^{2}+U_{2}{}^{4}-3U_{4}U_{2}{}^{2})+2U_{6}U_{2}{}^{2}]\cdot P_{1}^{2}\\ & +U_{2}{}^{3}(2U_{4}{}^{2}-U_{4}U_{2}{}^{2})-U_{6}U_{2}{}^{4}, \end{array}$$

By solving the aforementioned equation, we can obtain the second closed-form estimate for *k* and *µ* derived in this paper, as presented below:

(20) $$\left\{\begin{array}{l} {\hat{k}}_{A}=\frac{{\hat{P}}_{1}}{U_{2}-{\hat{P}}_{1}}\\ {\hat{\mu }}_{A}=\frac{U_{2}^{2}-{\hat{P}}_{1}^{2}}{U_{4}-U_{2}^{2}} \end{array}\right.,{\hat{P}}_{1}=\frac{-b+\frac{2AE}{B}-\sqrt{\frac{2B}{A}}}{4a}$$

Subsequently, we can derive the third closed-form estimate for *k* and *µ* in this paper, as shown below:

(21) $$\left\{\begin{array}{l} {\hat{k}}_{B}=\frac{{\hat{P}}_{1}}{U_{2}-{\hat{P}}_{1}}\\ {\hat{\mu }}_{B}=\frac{U_{2}^{2}-{\hat{P}}_{1}^{2}}{U_{4}-U_{2}^{2}} \end{array}\right.,{\hat{P}}_{1}=\frac{-b+\sqrt{D}}{4a}\;$$

**Figure 1:**
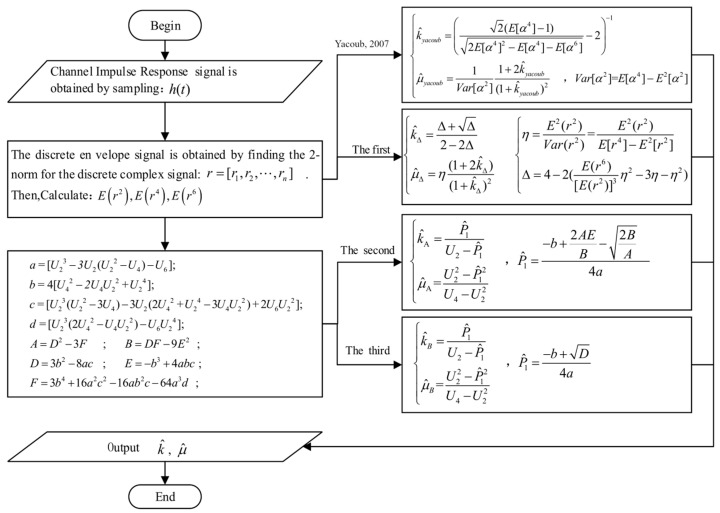
Flow chart of the parameter estimation [24].

The parameters in the model satisfy the following relationship:

(22) $$\begin{array}{l} a=[U_{2}{}^{3}-3U_{2}(U_{2}{}^{2}-U_{4})-U_{6}],\\ b=4[U_{4}{}^{2}-2U_{4}U_{2}{}^{2}+U_{2}{}^{4}],\\ c=[U_{2}{}^{3}(U_{2}{}^{2}-3U_{4})-3U_{2}(2U_{4}{}^{2}+U_{2}{}^{4}-3U_{4}U_{2}{}^{2})+2U_{6}U_{2}{}^{2}],\\ d=[U_{2}{}^{3}(2U_{4}{}^{2}-U_{4}U_{2}{}^{2})-U_{6}U_{2}{}^{4}],\\ A=D^{2}-3F,\;\;B=DF-9E^{2},\\ D=3b^{2}-8ac,\;\;E=-b^{3}+4abc\;,\\ F=3b^{4}+16a^{2}c^{2}-16ab^{2}c-64a^{3}d, \end{array}$$

and we summarize the estimation process of each estimator as below:

In Figure 1, the input to the estimator is the channel envelope data *r*, which consists of *N* sample points. At a certain moment, the instantaneous envelope *r* is the superposition of the useful signal and noise, which are temporarily indistinguishable. Consequently, in practical applications, it is necessary to filter the received signals. However, our estimator is also applicable to the study of noisy environments. In cases where the input signal is represented by instantaneous signal-to-noise ratios (SNRs) γ, the estimator proposed in this study remains equally valid and applicable.

In signal measurement experiments conducted in practical scenarios, the transmitter sends a pseudorandom sequence, and the channel information is obtained by correlation operating the received data with the known transmitted pseudorandom sequence. The calculation process for the CFR is denoted as $\hat{H}(f)=Y(f)\cdot conj(X(f))/|X(f){|}^{2}$, where X(f) represents the transmitted frequency domain signal (PN sequence), Y(f) denotes the down-converted IQ signal of the received frequency domain signal, and conj(⋅) signifies the complex conjugate. At this point, $\hat{H}(f)$ encompasses the transmitter system response $H_{T}(f)$, free-space channel response $H_{C}(f)$, and receiver system response $H_{R}(f)$. The noise of the transmitter system is contained within $H_{T}(f)$, the noise in the propagation medium is contained within $H_{C}(f)$, and the noise of the receiver system is contained within $H_{R}(f)$. The channel system can be represented as $Y(f)=H_{T}(f)\cdot H_{C}(f)\cdot H_{R}(f)\cdot X(f)$. To further calibrate the system, the transmitter and receiver are directly connected via a high-frequency cable to obtain the system response, where the received frequency domain signal is $Y_{sys}(f)=H_{T}(f)\cdot H_{R}(f)\cdot X(f)$. Consequently, the calibrated transmitter and receiver channel frequency response is denoted as ${\hat{H}}_{C}(f)=Y(f)/Y_{ysy}(f)$. Substituting the calculation process of the CFR, the calibrated transmitter and receiver channel frequency response is represented as ${\hat{H}}_{C}(f)=\hat{H}(f)\cdot X(f)/Y_{ysy}(f)$. Performing the inverse Fourier transform on ${\hat{H}}_{C}(f)$ results in the calibrated channel impulse response $\hat{h}(t)$, which is a discrete variable. By taking the magnitude of $\hat{h}(t)$, the discrete envelope signal $r=[r_{1},r_{2},r_{3},\cdots ,r_{n}]$ can be obtained. By calculating the second-, fourth-, and sixth-order moments of the envelope signal and incorporating them into the estimation process depicted in Figure 1, the *k* and *μ* parameters can be estimated.

## 4. Performance Analysis of Closed-Form Estimator

### 4.1. Computational Complexity Analysis of Closed-Form Estimator

Algorithmic complexity is a criterion for evaluating the execution efficiency of an effective algorithm. When the sample size is *N*, the total number of basic operations performed by different algorithms is denoted as time complexity, while the required memory space is denoted as space complexity. Necessary procedures that both types of complexities need to address are not considered in the comparison of complexities. We present the complexity analysis of four closed-form solutions in Table 2:

In Table 2, $O_{1}(\cdot )$ represents the number of basic operations required for computing other variables (excluding *k* and *μ*) before the closed-form calculation. For example, the $O_{1}(\cdot )$ value for Estimation Method $\hat{k}\text{-}{\hat{\mu }}_{\Delta }$ indicates the complexity of solving for variable Var, η, Δ; similarly, the $O_{1}(\cdot )$ value for Estimation Method $\hat{k}\text{-}{\hat{\mu }}_{B}$ also represents the complexity of solving for variable *a*, *b*, *c*, *D*, *P*_1_. $O_{2}(\cdot )$ denotes the complexity of the *k* and μ estimation functions, such as the closed-form expressions for Method $\hat{k}\text{-}{\hat{\mu }}_{Yacoub}$ given by Equations (6) and (7). $O_{3}(\cdot )$ refers to the memory space required to store temporary variables during the estimation process. For instance, Estimation Method $\hat{k}\text{-}{\hat{\mu }}_{Yacoub}$ only needs to store the variance operator *Var*[·], thus $O_{3}(\cdot )=1$. Since each moment estimation requires the calculation of the second-order, fourth-order, and sixth-order moments of the envelope, the computational cost of $E(r^{2}),E(r^{4}),E(r^{6})$ is not included in the complexity analysis.

To further analyze time complexity, we employ the Monte Carlo method to generate channel data following the kappa-mu distribution and compare estimation efficiency based on the time taken for the estimation. As shown in Figure 1, we use the channel impulse response *h*(*t*) as the data sample. The channel data generated using the Monte Carlo method in this section does not include noise.

We set the sample size to 10,000, with each round of estimation experiments using the same data. The efficiency of the estimator is evaluated based on the total runtime of multiple estimations, and the runtime is recorded in Table 3.

As can be seen from Table 2 and Table 3, Estimation Method $\hat{k}\text{-}{\hat{\mu }}_{Yacoub}$ has the optimal complexity, followed by Method $\hat{k}\text{-}{\hat{\mu }}_{\Delta }$, while Method $\hat{k}\text{-}{\hat{\mu }}_{A}$ is the least efficient. However, in the authors’ numerous simulation studies, it was found that when the estimation error of Method $E(r^{2}),E(r^{4}),E(r^{6})$ is relatively large, the estimator $\hat{k}\text{-}{\hat{\mu }}_{Yacoub}$ may produce *k* and *μ* values that are less than zero or complex numbers, which can be considered erroneous estimates. In practical applications, repeated estimations may generally consume more time.

### 4.2. The Simulation for Estimator Accuracy Analysis

Due to the probability density function (PDF) of the *κ-μ* fading channel model being controlled by the model parameters *κ* and *μ*, existing research has not yet been able to explain the conversion relationship between these two parameters. Consequently, it is not possible to evaluate the accuracy of the estimation algorithm using the deviation distance $\|(k,\mu )-(\hat{k},\hat{\mu }){\|}_{2}$ between the theoretical and estimated values. Therefore, this paper employs the error $\|f_{PDF}(k,\mu )-f_{PDF}(\hat{k},\hat{\mu }){\|}_{2}$ between the theoretical PDF curve and the estimated PDF curve, as well as the root mean square error (RMSE), to evaluate the estimation accuracy of the algorithm. The RMSE reflects the degree to which a set of data deviates from the true data. As the RMSE approaches 0, it indicates a smaller deviation between the theoretical PDF curve and the estimated PDF curve, resulting in higher estimation accuracy. The mathematical expression for RMSE is given as $\sqrt{\sum_{1}^{n}{\left(f_{PDF}(k,\mu ,r_{i})-f_{PDF}(\hat{k},\hat{\mu },r_{i})\right)}^{2}/n}$, where $r_{i}∼r_{n}$ represents the minimum and maximum values of the envelope. In the experiments of this paper, the gradient $\Delta r=r_{i+1}-r_{i}$ is 0.01. In the equation, $f_{PDF}(k,\mu ,r_{i})$ is the theoretical probability (assumed to be $P_{i}$) when the envelope is equal to $r_{i}$, and $f_{PDF}(\hat{k},\hat{\mu },r_{i})$ is the estimated probability (assumed to be ${\hat{P}}_{i}$) when the envelope is equal to $r_{i}$. The calculation method of RMSE can be expanded as ${\left[\left({\left(P_{1}-{\hat{P}}_{1}\right)}^{2}+{\left(P_{2}-{\hat{P}}_{2}\right)}^{2}+\cdots +{\left(P_{i}-{\hat{P}}_{i}\right)}^{2}+\cdots +{\left(P_{n}-{\hat{P}}_{n}\right)}^{2}\right)/n\right]}^{\;0.5}$.

**Figure 2:**
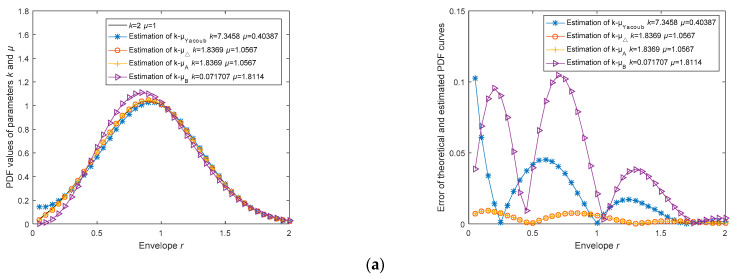

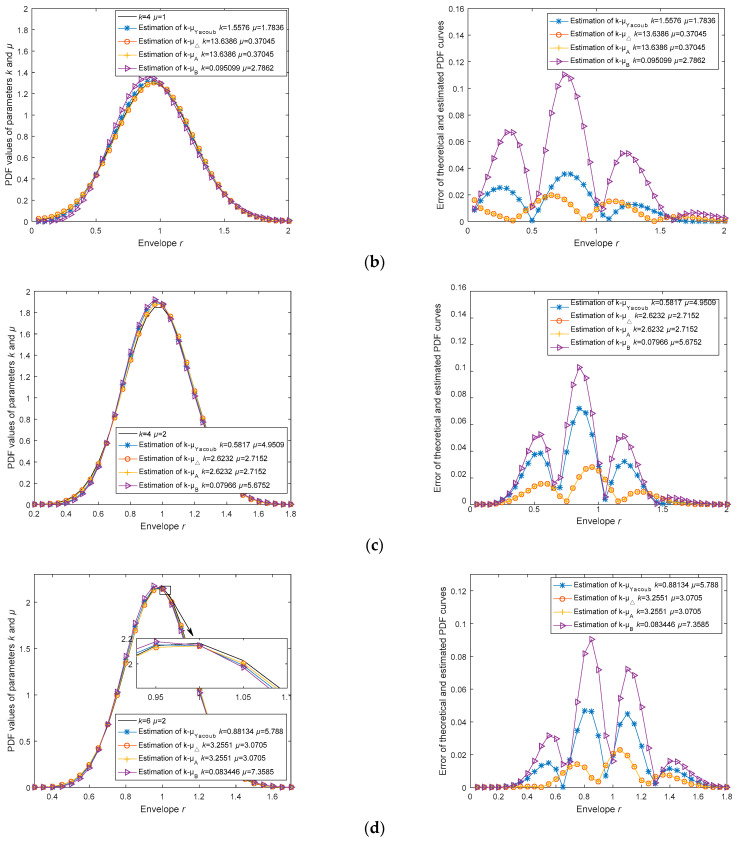
Estimation accuracy analysis of *k*, *µ* parameter estimation methods based on Monte Carlo method generated *k-µ* channel data; (**a**) Comparison and error of theoretical PDF and estimated PDF curves at *k* = 2, *µ* = 1; (**b**) Comparison and error of theoretical PDF and estimated PDF curves at *k* = 4, *µ* = 1; (**c**) Comparison and error of theoretical PDF and estimated PDF curves at *k* = 4, *µ* = 2; (**d**) Comparison and error of theoretical PDF and estimated PDF curves at *k* = 6, *µ* = 2.

Simulation experiment samples were generated using the Monte Carlo method, producing channel data that follows the *κ-μ* distribution. In each estimation experiment, four estimators employed the same set of generated channel data, with a sample size of 10,000. Four simulation experiments were conducted with the following parameter settings: *κ* = 2 and *μ* = 1; *κ* = 4 and *μ* = 1; *κ* = 4 and *μ* = 2; and *κ* = 6 and *μ* = 2. The estimated PDF curves were plotted based on the estimation results and compared with the theoretical PDF curves. The comparison and error plots are illustrated in Figure 2. The estimated values and root mean square errors were rounded to the 12th decimal place and recorded in Table 4.

In Figure 2, the left column of the plots demonstrates the comparison between the PDF curves calculated from the estimated *k* and *μ* parameters and the theoretical PDF curves. The right column of plots shows the errors between the four estimated PDF curves and the theoretical PDF curves. In Table 3, RMSE*_pdf_* refers to the root mean square error between the PDF curve and the theoretical PDF curve, while the last column displays the ranking of estimation accuracy for each estimator in each experiment.

Throughout numerous experiments conducted during the research process, we discovered that the *k, μ*, and RMSE*_pdf_* values for estimators $\hat{k}\text{-}{\hat{\mu }}_{\Delta }$ and $\hat{k}\text{-}{\hat{\mu }}_{A}$ were nearly identical, with differences only appearing beyond the seventh decimal place. Consequently, we consider them to have the same estimation accuracy.

Meanwhile, in multiple experiments, Estimator $\hat{k}\text{-}{\hat{\mu }}_{Yacoub}$ frequently exhibits instances of negative or complex values for *k* and *µ*, which we consider to be incorrect estimations. The cause of this phenomenon is the lack of stability in Estimator $\hat{k}\text{-}{\hat{\mu }}_{Yacoub}$, and there are three factors contributing to its instability:

Firstly, in Equation (6) ${\hat{k}}_{yacoub}$, the denominator of the term $2E{\left[\alpha^{4}\right]}^{2}-E[\alpha^{4}]-E[\alpha^{6}]$ often becomes negative, resulting in complex-valued estimates.

Secondly, Estimator ${\hat{k}}_{yacoub}$ violates two principles of numerical computation that should be adhered to. Ideally, the subtraction of two numbers with close values should be avoided. However, in the denominator of term ${\hat{k}}_{yacoub}$ in Equation (6), the values of $E[\alpha^{6}]$ are very close to that of $2E{\left[\alpha^{4}\right]}^{2}-E[\alpha^{4}]$.

Last, it is preferable to avoid dividing a large number by a small one. Nonetheless, in Equation (6) ${\hat{k}}_{yacoub}$, the numerator $\sqrt{2}(E[\alpha^{4}]-1)$ is several orders of magnitude larger than the denominator $\sqrt{2E{\left[\alpha^{4}\right]}^{2}-E[\alpha^{4}]-E[\alpha^{6}]}$.

Therefore, under the experimental scenarios presented in Table 4, Estimator $\hat{k}\text{-}{\hat{\mu }}_{\Delta }$ and $\hat{k}\text{-}{\hat{\mu }}_{A}$ outperforms other estimators. Moreover, the computation time of Estimator $\hat{k}\text{-}{\hat{\mu }}_{\Delta }$ is smaller than that of Estimator $\hat{k}\text{-}{\hat{\mu }}_{A}$, making Estimator $\hat{k}\text{-}{\hat{\mu }}_{\Delta }$ the most optimal choice.

**Figure 3:**
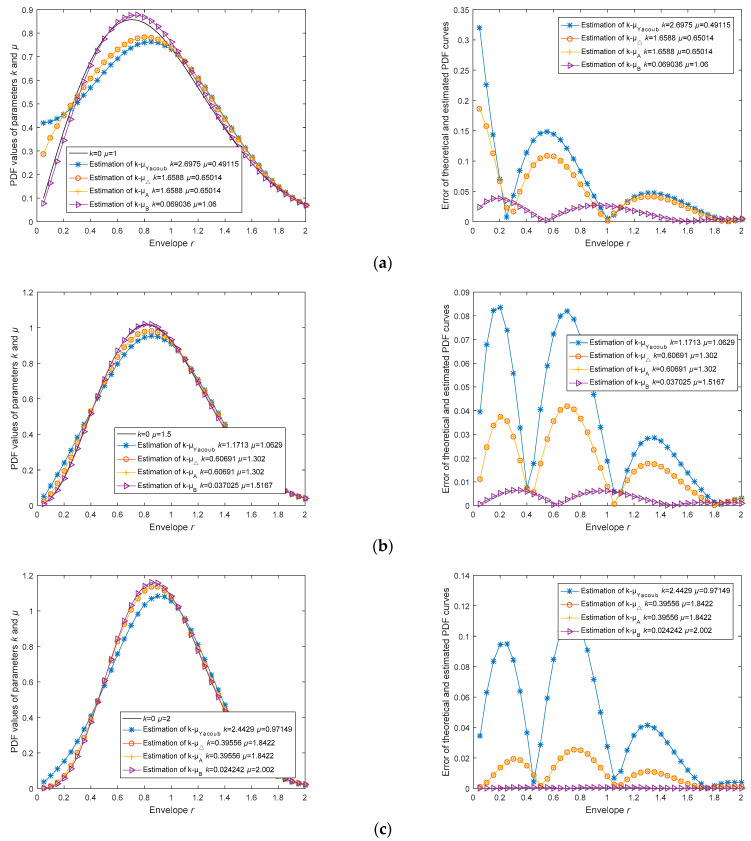
Estimation accuracy analysis of *k*, *µ* parameter estimation algorithm based on Monte Carlo method generated *k-µ* channel data; (**a**) Comparison the error of theoretical PDF and estimated PDF curves at *k* = 0, *µ* = 1; (**b**) Comparison the error of theoretical PDF and estimated PDF curves at *k* = 0, *µ* = 1.5; (**c**) Comparison the error of theoretical PDF and estimated PDF curves at *k* = 0, *µ* = 2.

Additionally, we conducted another set of comparative experiments. Assuming *k* = 0 and *µ* taking values of 1, 1.5, and 2, we generated channel data using the Monte Carlo method. In each estimation experiment, the four estimators used the same data samples, with a sample size of 10,000. The estimated PDF curves, theoretical PDF curves, and their errors $\|f_{PDF}(k,\mu )-f_{PDF}(\hat{k},\hat{\mu }){\|}_{2}$ are depicted in Figure 3. Furthermore, the estimation results and root mean square errors (RMSE) are recorded in Table 5.

In Figure 3, the experiments for different *µ* values under the condition of *k* = 0 are presented. The left image displays a comparison between the estimated PDF curves and the theoretical PDF curves, while the right image illustrates the errors between the estimated PDF curves and the theoretical PDF curves. In Table 5, the RMSE*_pdf_* column shows the root mean square errors between the PDF curves and the theoretical PDF curves; values closer to 0 indicate higher estimation accuracy. The last column demonstrates the ranking of estimation accuracy for each estimator in every experiment.

In Table 5, we still observe that the *k, µ*, and RMSE*_pdf_* values of Estimator $\hat{k}\text{-}{\hat{\mu }}_{Yacoub}$ and $\hat{k}\text{-}{\hat{\mu }}_{Yacoub}$ are almost identical, with differences only occurring after the seventh decimal place. Consequently, we can assert that these two estimators have the same estimation accuracy, regardless of the variations in *k* and *µ*.

At the same time, Estimator $\hat{k}\text{-}{\hat{\mu }}_{Yacoub}$ continues to exhibit incorrect estimates with negative or complex values for *k* and *µ.* In cases of correct estimation, as shown in Table 5, the RMSE*_pdf_* of the estimator is sufficiently small.

From the last column in Table 5, it can be observed that under the condition of *k* = 0, Estimator $\hat{k}\text{-}{\hat{\mu }}_{B}$ has the best estimation accuracy, while Estimator $\hat{k}\text{-}{\hat{\mu }}_{Yacoub}$ has the worst. Therefore, Estimator $\hat{k}\text{-}{\hat{\mu }}_{Yacoub}$ is more suitable for situations with extremely poor channel quality, where there are numerous obstacles between the transmitting and receiving antennas and no line-of-sight component (*k* approaching 0).

In summary, through complexity analysis and simulation, we find that Estimator $\hat{k}\text{-}{\hat{\mu }}_{Yacoub}$ has the lowest estimation efficiency. When *k* approaches 0, using Estimator $\hat{k}\text{-}{\hat{\mu }}_{B}$ is the best choice; in other cases, Estimator $\hat{k}\text{-}{\hat{\mu }}_{\Delta }$ is the most optimal option.

### 4.3. Impact of SNR on Estimator Performance Analysis

Noise can cause an increase in the bias and variance of the estimators, thereby reducing the accuracy and efficiency of the estimation. In order to analyze the impact of noise on the accuracy of the estimators, we assume signal-to-noise ratios (SNRs) of 30 dB, 25 dB, 20 dB, and 15 dB and conduct simulation analysis under the assumption of channel parameters *k* = 3.5 and *μ* = 1.5. In this case, the envelope signal satisfies the relationship $r_{addNoise}=r_{k-\mu Signal}+r_{noise}$, $r_{k-\mu Signal}$ is generated by the Monte Carlo method, and $r_{noise}$ represents additive Gaussian white noise. For ease of comparison, the analysis results here are presented with a precision of four decimal places. In each experiment, the four estimators use the same sample data, and the sample size is 10,000.

In multiple simulation experiments, we find that when the SNR is 30 dB, the estimation method $\hat{k}\text{-}{\hat{\mu }}_{Yacoub}$ has about a 40% probability of not producing valid results; when the SNR is 25 dB, the estimation method $\hat{k}\text{-}{\hat{\mu }}_{Yacoub}$ has about a 60% probability of not producing valid results; when the SNR is 20 dB, the estimation method $\hat{k}\text{-}{\hat{\mu }}_{Yacoub}$ has about a 90% probability of not producing valid results; and when the SNR is 15 dB, the estimation method $\hat{k}\text{-}{\hat{\mu }}_{Yacoub}$ can no longer produce valid results. In all the above experiments, the channel parameter estimator we proposed demonstrated significantly lower error probabilities under the aforementioned signal-to-noise ratio levels, indicating its robustness in various noise conditions. In the figures below, we present the valid estimation results.

**Figure 4:**
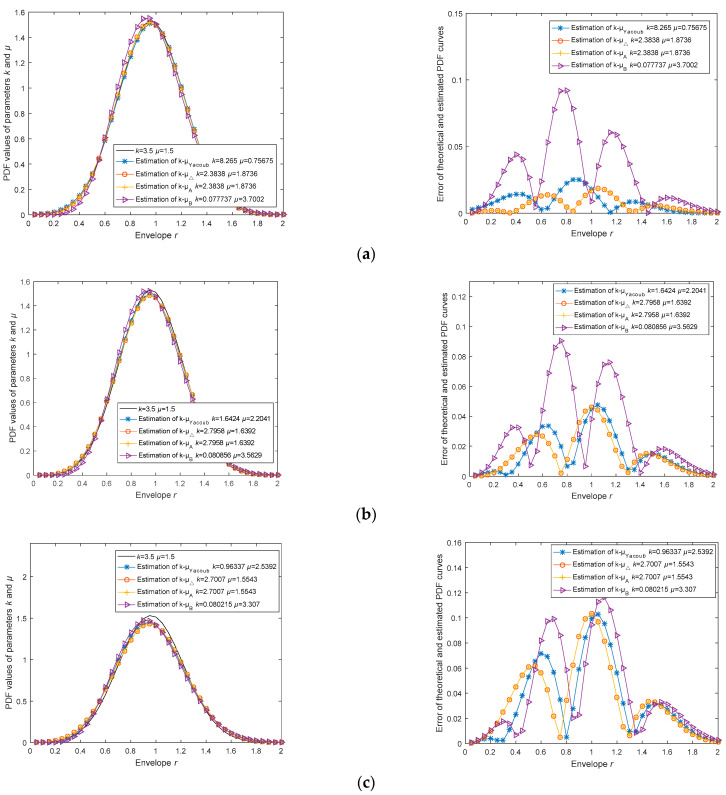

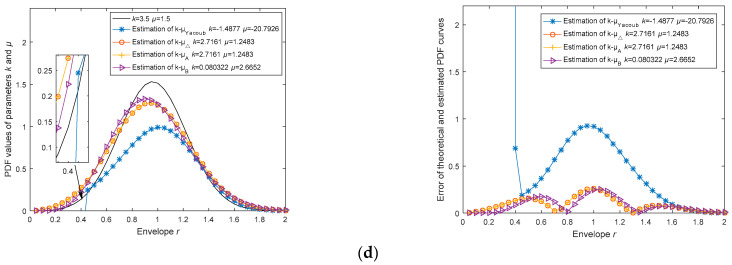
Impact analysis of estimator performance based on different SNR conditions; (**a**) Error comparison of theoretical and estimated PDF curves at *k* = 3.5, *μ* = 1.5, SNR = 30 dB; (**b**) Error comparison of theoretical and estimated PDF curves at *k* = 3.5, *μ* = 1.5, SNR = 25 dB; (**c**) Error comparison of theoretical and estimated PDF curves at *k* = 3.5, *μ* = 1.5, SNR = 20 dB; (**d**) Error comparison of theoretical and estimated PDF curves at *k* = 3.5, *μ* = 1.5, SNR = 15 dB.

From Table 6, we can see that the parameters *k* and *µ* have already become less than 0, and the $\hat{k}\text{-}{\hat{\mu }}_{Yacoub}$ estimation method can no longer produce valid results. Moreover, in Figure 4d, it can be observed that the curve obtained from the $\hat{k}\text{-}{\hat{\mu }}_{Yacoub}$ estimation method has been completely deformed. This indicates that the $\hat{k}\text{-}{\hat{\mu }}_{Yacoub}$ method will fail when the noise interference is significant.

From the RMSE*_pdf_* column in Table 6, it can be seen that as the SNR decreases, the estimation errors of the estimators gradually increase. When the SNR drops to 15 dB, the estimation errors of all estimators are essentially unacceptable. Therefore, we can draw the conclusion that our proposed estimators have limitations in their performance when the SNR is low. When using the estimators proposed in this paper as well as Yacoub’s estimator, it is necessary to first filter out the noise or increase the signal gain. Furthermore, as the SNR decreases, the RMSE values of all estimators gradually increase. At the 15 dB level, $\hat{k}\text{-}{\hat{\mu }}_{Yacoub}$ estimator cannot be computed, and with its higher estimation error probability, it becomes unsuitable for practical applications. Moreover, although the RMSE of the $\hat{k}\text{-}{\hat{\mu }}_{B}$ estimator is higher than that of the $\hat{k}\text{-}{\hat{\mu }}_{\Delta }$/$\hat{k}\text{-}{\hat{\mu }}_{A}$ estimator under higher SNR conditions, as the SNR decreases, the performance of the $\hat{k}\text{-}{\hat{\mu }}_{B}$ estimator declines more slowly than that of the $\hat{k}\text{-}{\hat{\mu }}_{\Delta }$/$\hat{k}\text{-}{\hat{\mu }}_{A}$ estimator. Therefore, at 15 dB, the RMSE performance of the $\hat{k}\text{-}{\hat{\mu }}_{B}$ estimator is better than that of the $\hat{k}\text{-}{\hat{\mu }}_{\Delta }$/$\hat{k}\text{-}{\hat{\mu }}_{A}$ estimator, which intuitively reflects the correct trend of estimation accuracy changes for different channel parameter estimators under different SNR conditions. In the methods proposed in this paper, the $\hat{k}\text{-}{\hat{\mu }}_{\Delta }$/$\hat{k}\text{-}{\hat{\mu }}_{A}$ estimator is used for communication scenarios with better channel conditions, while the $\hat{k}\text{-}{\hat{\mu }}_{B}$ estimator is suitable for non-line-of-sight (NLOS) scenarios with poorer channel conditions and lower received SNR. This is consistent with the conclusions reached.

### 4.4. Impact of Pilot Signal on Estimator Performance Analysis

In general, the amount of information required by the pilot signal depends on the specific estimation methods and the channel environment. The impact of the pilot signal on the estimation performance also varies with different estimation algorithms and channel conditions. Basically, a higher pilot signal power or density can lead to better channel estimation accuracy, but also at the cost of higher overhead and decreased spectral efficiency. On the other hand, a lower pilot signal power or density can result in lower accuracy and higher estimation error, especially in fast-varying channels.

Consequently, it is indeed true that with a longer pilot signal, more samples can be obtained, and as the sample size increases, the estimations of *k* and *μ* parameters will approach unbiasedness and become more accurate. In practical applications, extending the pilot signal duration and increasing the sampling rate can enhance estimation accuracy. However, this comes at the cost of consuming more communication resources, which in turn results in a reduced data rate for wireless communication. It is essential to weigh these trade-offs carefully.

To address this question, we conducted related parameter measurements and fitting simulation experiments in an indoor environment. The wireless communication scenario parameters are set as follows: The operating frequency band is the C-band (5 GHz); assuming a Rician channel with a channel length of 20, the transmitter employs a pilot PN sequence of 2048 symbols (25% of the total amount of transmitted data), BPSK modulation, and a code rate of 20 Mbps transmission scheme. On the receiver. At the receiving end, we used a receiving demodulation and processing device equipped with high-speed sampling, fiber-optic broadband transmission, and high-speed storage, achieving a transmission rate of 5 Gbps. The sampling frequency can reach up to 500 Msps.

**Figure 5:**
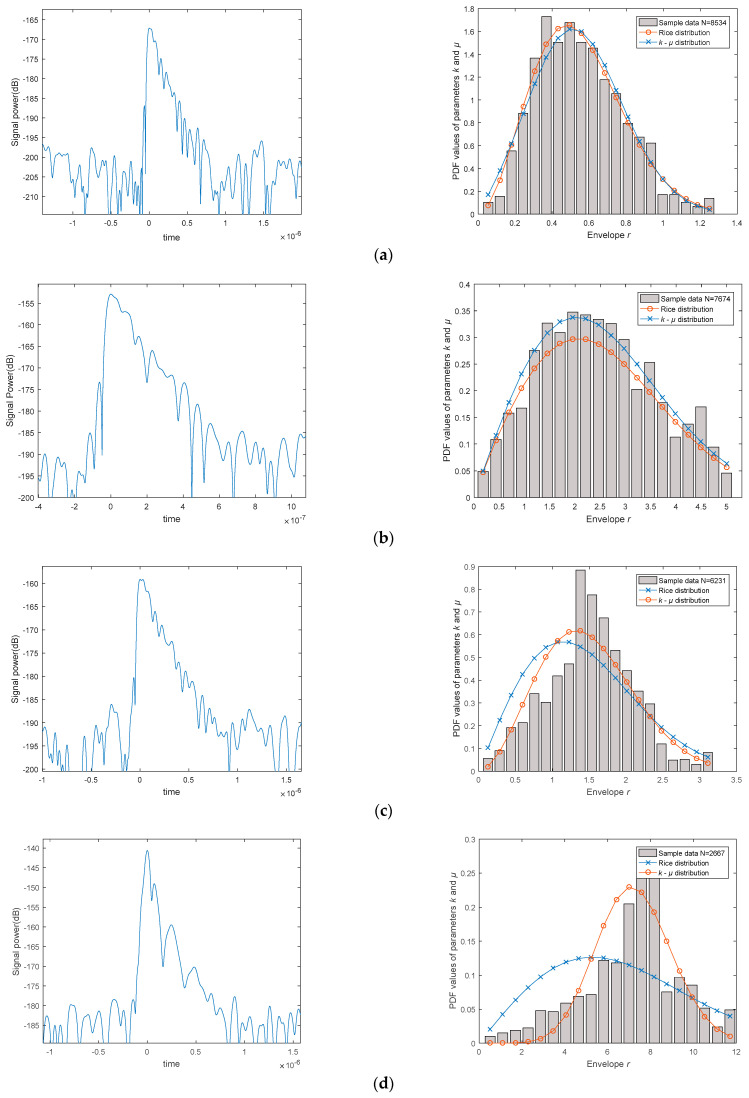

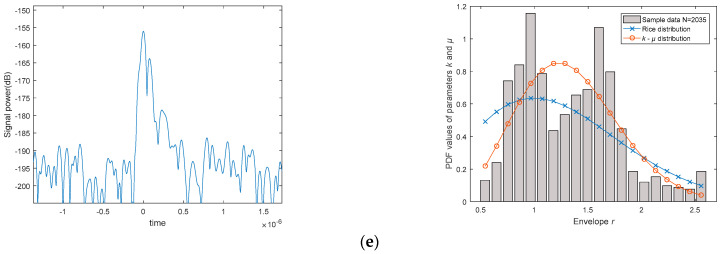
Impact analysis of estimator performance based on different SNR conditions: (**a**) The power delay spectrum and PDF values of parameters *k* and *µ* when *N* = 8500; (**b**) The power delay spectrum and PDF values of parameters *k* and *µ* when *N* = 8000; (**c**) The power delay spectrum and PDF values of parameters *k* and *µ* when *N* = 5000; (**d**) The power delay spectrum and PDF values of parameters *k* and *µ* when *N* = 2600; (**e**) The power delay spectrum and PDF values of parameters *k* and *µ* when *N* = 1000.

The left column in Figure 5 shows the time on the x-axis and signal power on the y-axis (within a one-second acquisition period, we detected 10 instances of robust signal waves; however, only a single representative example is displayed here). On the right column, we estimate the received signal, where the gray area represents the frequency histogram of envelope *r*, and the two curves are estimation curves, with the *x*-axis representing the normalized envelope and the *y*-axis representing the probability. In the right column, using the estimator proposed in this paper and defining *µ* = 1, we can obtain the estimated curve—“Rice distribution” (by taking advantage of the *k-µ* distribution transforming into the Rice distribution when *µ* = 1). Using the estimator without restricting the values of *k* and *μ*, we can obtain the curve—“*k-μ* distribution”. As can be seen from the graphs, when the sample size is sufficient, the *k-µ* distribution approximates the Rice distribution. When the sample size is not enough, the *k-µ* distribution estimator exhibits excellent adaptability.

As Figure 5 indicates, as the number of sampling points increases, the *k-µ* distribution becomes more closely aligned with the actual probability distribution, and the greater the number of samples, the more evident the alignment. In this experimental scenario, when the number of samples *N* obtained from pilot signal sampling is greater than 8000, the PDF estimation curve generated by the sample points essentially matches the histogram and theoretical curve. Consequently, a consistent correlation between the theoretical analysis and experimental measurement results concerning the impact of the pilot signal on estimator performance is observed.

## 5. Conclusions

The paper introduces a novel and efficient methodology for estimating multiple parameters in the *k-µ* fading channel model. Two sets of solutions are derived using moment-generating functions based on second-, fourth-, and sixth-order moments. Three sets of closed-form solutions are designed for estimating the *k* and *µ* parameters based on the previously derived solutions. Performance analysis of the proposed parameter estimators using simulation results demonstrates strong consistency between theoretical and estimated values. The proposed parameter estimators address some of the shortcomings of existing estimators through simulation and comparison, including large estimation errors, susceptibility to errors, and fragility to noise interference. The estimators can calculate *k* and *µ* parameters based on the envelope and SNR signals. The work presents a mathematically tractable framework for estimating characteristic parameters of wireless channels in small-scale environments. It is expected to allow for real-time and accurate analysis of complex scenarios, such as moving targets, time-varying conditions, or non-line-of-sight (NLOS) extreme communication, based on *k-µ* fading channel model parameters. Simulation results demonstrate that the proposed parameter estimator $\hat{k}\text{-}{\hat{\mu }}_{\Delta }$ is appropriate for low latency and limited resource overhead communication scenarios, and the other estimator $\hat{k}\text{-}{\hat{\mu }}_{B}$ is suitable for scenarios with poor channel quality, numerous obstacles between antennas, or no LOS communication path available (*k* approaches zero, NLOS communication scenarios).

## Figures and Tables

**Table 1:** The development process of the fading channel model.

Model Number	Model Type	Degradation to Other Models	Reference
1	One-sided Gaussian		[5]
2	Rayleigh distribution		[6]
3	Rician distribution	2	[7]
4	Nakagami-*m* distribution	2	[8]
5	Hoyt (Nakagami-q) distribution		[9]
6	*k*-*µ* distribution	1, 2, 3, 4	[10]
7	*k*-*µ* shadowed fading	1, 2, 3, 4, 5, 6, 7	[4]

**Table 2:** Algorithm complexity characteristics table.

Estimator Name	Time Complexity T(n)	Space Complexity S(n)	Estimation Efficiency
$\hat{k}\text{-}{\hat{\mu }}_{Yacoub}$ [24]	$O_{1}(2)+O_{2}(16)=O(n^{0})$	$O_{3}(1)=O(n^{0})$	1
$\hat{k}\text{-}{\hat{\mu }}_{\Delta }$	$O_{1}(15)+O_{2}(10)=O(n^{0})$	$O_{3}(3)=O(n^{0})$	2
$\hat{k}\text{-}{\hat{\mu }}_{A}$	$O_{1}(101)+O_{2}(8)=O(n^{0})$	$O_{3}(10)=O(n^{0})$	4
$\hat{k}\text{-}{\hat{\mu }}_{B}$	$O_{1}(50)+O_{2}(8)=O(n^{0})$	$O_{3}(5)=O(n^{0})$	3

**Table 3:** Total runtime of the estimator characteristics table.

Estimator Name	Number of Samples 10,000		Ranking
	Estimated 1 Time	Estimated 50 Time	
$\hat{k}\text{-}{\hat{\mu }}_{Yacoub}$ [24]	0.001372	0.017895	1st
$\hat{k}\text{-}{\hat{\mu }}_{\Delta }$	0.001481	0.018135	2nd
$\hat{k}\text{-}{\hat{\mu }}_{A}$	0.004415	0.048401	4th
$\hat{k}\text{-}{\hat{\mu }}_{B}$	0.002582	0.028465	3rd

**Table 4:** Performance comparison table of estimators for *k*-*µ* fading channels when *µ* = 1.

Number	Factor		*k*	*µ*	RMSE*_pdf_*	Top	Figure
1	Truth value		2	1	/	/	Figure 2a
	Estimation Method	$\hat{k}\text{-}{\hat{\mu }}_{Yacoub}$ [24]	7.345803258921	0.403866253717	0.028617213697	3rd	
		$\hat{k}\text{-}{\hat{\mu }}_{\Delta }$	1.836935521234	1.056679155891	0.004732242111	1st	
		$\hat{k}\text{-}{\hat{\mu }}_{A}$	1.836935521235	1.056679155891	0.004732242111	1st	
		$\hat{k}\text{-}{\hat{\mu }}_{B}$	0.071707062788	1.811409799105	0.053510658991	4th	
2	Truth value		4	1	/	/	Figure 2b
	Estimation Method	$\hat{k}\text{-}{\hat{\mu }}_{Yacoub}$ [24]	1.557613414953	1.783643777602	0.016863852210	3rd	
		$\hat{k}\text{-}{\hat{\mu }}_{\Delta }$	13.638564206393	0.370451235968	0.009715164656	1st	
		$\hat{k}\text{-}{\hat{\mu }}_{A}$	13.638564206103	0.370451235975	0.009715164656	1st	
		$\hat{k}\text{-}{\hat{\mu }}_{B}$	0.095099435442	2.786154089865	0.048716935761	4th	
3	Truth value		4	2	/	/	Figure 2c
	Estimation Method	$\hat{k}\text{-}{\hat{\mu }}_{Yacoub}$ [24]	0.581701867497	4.950940693780	0.026659232023	3rd	
		$\hat{k}\text{-}{\hat{\mu }}_{\Delta }$	2.623205011929	2.715157714236	0.010815350647	1st	
		$\hat{k}\text{-}{\hat{\mu }}_{A}$	2.623205011909	2.715157714249	0.010815350647	1st	
		$\hat{k}\text{-}{\hat{\mu }}_{B}$	0.079660408693	5.675180409116	0.037623713820	4th	
4	Truth value		6	2	/	/	Figure 2d
	Estimation Method	$\hat{k}\text{-}{\hat{\mu }}_{Yacoub}$ [24]	0.881337139779	5.787975718336	0.019774889712	3rd	
		$\hat{k}\text{-}{\hat{\mu }}_{\Delta }$	3.255105553371	3.070458936537	0.008496533664	1st	
		$\hat{k}\text{-}{\hat{\mu }}_{A}$	3.255105553489	3.070458936463	0.008496533664	1st	
		$\hat{k}\text{-}{\hat{\mu }}_{B}$	0.083446313862	7.358476845620	0.034970287599	4th	

**Table 5:** Performance comparison table of estimators for *k*-*µ* fading channels when *k* = 0.

Number	Factor		*k*	*µ*	RMSE*_pdf_*	Top	Figure
1	Truth value		0	1	/	/	Figure 3a
	Estimation Method	$\hat{k}\text{-}{\hat{\mu }}_{Yacoub}$ [24]	2.697529972710	0.491148658790	0.094653847993	4th	
		$\hat{k}\text{-}{\hat{\mu }}_{\Delta }$	1.658816467801	0.650138179259	0.065828663572	2nd	
		$\hat{k}\text{-}{\hat{\mu }}_{A}$	1.658816467801	0.650138179259	0.065828663572	2nd	
		$\hat{k}\text{-}{\hat{\mu }}_{B}$	0.069035813789	1.060038794578	0.019338029254	1st	
2	Truth value		0	1.5	/	/	Figure 3b
	Estimation Method	$\hat{k}\text{-}{\hat{\mu }}_{Yacoub}$ [24]	1.171262682065	1.062944179515	0.043970366233	4th	
		$\hat{k}\text{-}{\hat{\mu }}_{\Delta }$	0.606908589722	1.302013307991	0.021477554324	2nd	
		$\hat{k}\text{-}{\hat{\mu }}_{A}$	0.606908589723	1.302013307991	0.021477554324	2nd	
		$\hat{k}\text{-}{\hat{\mu }}_{B}$	0.037025064065	1.516708286752	0.003692816335	1st	
3	Truth value		0	2	/	/	Figure 3c
	Estimation Method	$\hat{k}\text{-}{\hat{\mu }}_{Yacoub}$ [24]	2.442853784456	0.971494141133	0.056600362496	4th	
		$\hat{k}\text{-}{\hat{\mu }}_{\Delta }$	0.395558380575	1.842234181266	0.012151341100	2nd	
		$\hat{k}\text{-}{\hat{\mu }}_{A}$	0.395558380576	1.842234181266	0.012151341100	2nd	
		$\hat{k}\text{-}{\hat{\mu }}_{B}$	0.024242478356	2.002043886281	0.000434639810	1st	

**Table 6:** The comparison of estimation results under different SNR conditions.

*SNR* (dB)	Factor		*k*	*µ*	RMSE*_pdf_*	Top	Figure
	Truth Value		3.5	1.5			
30 dB	Estimation Method	$\hat{k}\text{-}{\hat{\mu }}_{Yacoub}$	8.2650	0.7567	0.0109	/	Figure 4a
		$\hat{k}\text{-}{\hat{\mu }}_{\Delta }$/$\hat{k}\text{-}{\hat{\mu }}_{A}$	2.3838	1.8736	0.0081	1	
		$\hat{k}\text{-}{\hat{\mu }}_{B}$	0.0777	3.7002	0.0387	2	
25 dB	Estimation Method	$\hat{k}\text{-}{\hat{\mu }}_{Yacoub}$	1.6424	2.2041	0.0205	/	Figure 4b
		$\hat{k}\text{-}{\hat{\mu }}_{\Delta }$/$\hat{k}\text{-}{\hat{\mu }}_{A}$	2.7958	1.6392	0.0197	1	
		$\hat{k}\text{-}{\hat{\mu }}_{B}$	0.0809	3.5629	0.0402	2	
20 dB	Estimation Method	$\hat{k}\text{-}{\hat{\mu }}_{Yacoub}$	2.8294	1.4664	0.0554	/	Figure 4c
		$\hat{k}\text{-}{\hat{\mu }}_{\Delta }$/$\hat{k}\text{-}{\hat{\mu }}_{A}$	5.7361	0.8852	0.0557	1	
		$\hat{k}\text{-}{\hat{\mu }}_{B}$	0.0903	3.1984	0.0605	2	
15 dB	Estimation Method	$\hat{k}\text{-}{\hat{\mu }}_{Yacoub}$	−1.4877	−20.7926	/	/	Figure 4d
		$\hat{k}\text{-}{\hat{\mu }}_{\Delta }$/$\hat{k}\text{-}{\hat{\mu }}_{A}$	2.7161	1.2483	0.1153	2	
		$\hat{k}\text{-}{\hat{\mu }}_{B}$	0.0803	2.6652	0.1129	1	

## Data Availability

Not applicable.

## Appendix A

The transformation form of confluent hypergeometric function given in [30]:

(A1) F11(α,ϒ;z)=ez⋅F11(ϒ−α,ϒ;−z)

The moment function of the *k*-*µ* distribution can be further expressed as:

(A2) E[rn]=E(r2)n⋅Γ(μ+n/2)⋅exp(−kμ)Γ(μ)⋅[(1+k)μ]n/2F11(μ+n/2,μ;kμ)

where *n* is the order of the moment function, Γ(⋅) is the gamma function, and Γ(n+1)=n!. F11(⋅,⋅ ; ⋅) is the *Kummer* confluent hypergeometric function, with the expressions ([30], Section 9.212, Equation (1)):

(A3) F11(α,β;x)=∑n=0∞(α)nxn(β)nn!,(α)n=1(α)(α+1)⋯(α+n−1)

Substituting the *gamma* function and the *Kummer* confluent hypergeometric function into Equation (A2), setting *n* to 2 and 4, and eliminating the gamma function term using the second-order moments compared to the fourth-order distance:

(A4) E(r2)E(r4)=E(r2)⋅Γ(μ+1)Γ(μ)⋅[(1+k)μ]1⋅F11(−1,μ;−kμ)E(r2)2⋅Γ(μ+2)Γ(μ)⋅[(1+k)μ]2⋅F11(−2,μ;−kμ)=μ![(1+k)μ]E(r2)(μ+1)!⋅F11(−1,μ;−kμ)F11(−2,μ;−kμ)=(1+k)μE(r2)(μ+1)⋅[1+(−1)μ(−kμ)1!+(−1)(0)μ(μ+1)(−kμ)22!+(−1)(0)(1)μ(μ+1)(μ+2)(−kμ)33!+(−1)(0)(1)(2)μ(μ+1)(μ+2)(μ+3)(−kμ)44!+⋯][1+(−2)μ(−kμ)1!+(−2)(−1)μ(μ+1)(−kμ)22!+(−2)(−1)(0)μ(μ+1)(μ+2)(−kμ)33!+(−2)(−1)(0)(1)μ(μ+1)(μ+2)(μ+3)(−kμ)44!+⋯]=(1+k)μE(r2)(μ+1)⋅(1+k)(μ+1)+2k(μ+1)+k2μ(μ+1)=1E(r2)⋅μ(1+k)2μ(1+k)2+1+2k

After organizing the above equations, the expression for the fourth-order moment is given by

(A5) $$E(r^{4})=E{\left(r^{2}\right)}^{2}\cdot \left(1+\frac{1+2k}{\mu {\left(1+k\right)}^{2}}\right)$$

Equivalently, we then set *n* to be 2 and 6, and using the second-order moments compared with the sixth-order distance to eliminate the *gamma* function term, we can obtain the ratio relationship between the second-order moments and the sixth-order moments as follows:

(A6) E(r2)E(r6)=E(r2)⋅Γ(μ+1)Γ(μ)⋅[(1+k)μ]1⋅F11(−1,μ;−kμ)E(r2)3⋅Γ(μ+3)Γ(μ)⋅[(1+k)μ]3⋅F11(−3,μ;−kμ)=μ![(1+k)μ]2E(r2)2(μ+2)!⋅F11(−1,μ;−kμ)F11(−3,μ;−kμ)=[(1+k)μ]2E(r2)2(μ+1)(μ+2)⋅[1+(−1)μ(−kμ)1!][1+(−3)μ(−kμ)1!+(−3)(−2)μ(μ+1)(−kμ)22!+(−3)(−2)(−1)μ(μ+1)(μ+2)(−kμ)33!]=1E(r2)2⋅μ3(1+k)3μ(μ+1)(μ+2)+3kμ(μ+1)(μ+2)+3(kμ)2(μ+2)+(kμ)3

After the organization of Equation (A6), the expression for the sixth-order moments can be equivalent to

(A7) $$E(r^{6})=E{\left(r^{2}\right)}^{3}\cdot \left(\frac{\mu (\mu +1)(\mu +2)+3k\mu (\mu +1)(\mu +2)+3{\left(k\mu \right)}^{2}(\mu +2)+{\left(k\mu \right)}^{3}}{{\left(1+k\right)}^{3}\mu^{3}}\right)$$

## Appendix B

The total LOS path energy of the *k*-*µ* distribution is now defined as *P*_1_ and the total NLOS path energy as *P*_2_. In the physical model of the *k*-*µ* distribution, we have the relationship $\sum_{1}^{n}\left(p_{i}^{2}+q_{i}^{2}\right)/\sum_{1}^{n}\left(x_{i}^{2}+y_{i}^{2}\right)=P_{1}/P_{2}$, the total received power $E(r^{2})=P_{1}+P_{2}$. As a result, the fourth-order moment function of the *k*-*µ* distribution (Equation (A5)) can be reformulated as:

(A8) $$\begin{array}{l} E(r^{4})=E{\left(r^{2}\right)}^{2}\cdot \left(1+\frac{1+2k}{\mu {\left(1+k\right)}^{2}}\right)={\left(P_{1}+P_{2}\right)}^{2}\left(\frac{\mu {\left(\frac{P_{1}+P_{2}}{P_{2}}\right)}^{2}+1+2\frac{P_{1}}{P_{2}}}{\mu {\left(\frac{P_{1}+P_{2}}{P_{2}}\right)}^{2}}\right)\\ =\frac{P_{2}^{2}\cdot \mu {\left(\frac{P_{1}+P_{2}}{P_{2}}\right)}^{2}+P_{2}^{2}\cdot 1+P_{2}^{2}\cdot 2\frac{P_{1}}{P_{2}}}{\mu }={\left(P_{1}+P_{2}\right)}^{2}+\frac{1}{\mu }(P_{2}^{2}+2P_{1}P_{2}+P_{1}^{2})-\frac{1}{\mu }P_{1}^{2}\\ =\frac{\mu +1}{\mu }U_{2}^{2}-\frac{1}{\mu }P_{1}^{2} \end{array}$$

Similarly, Equation (A7) in Appendix A, that is, the fourth-order moment function of the *k-µ* distribution, can be reformulated as:

(A9) $$\begin{array}{l} E(r^{6})=E{\left(r^{2}\right)}^{3}\cdot \left(\frac{\mu (\mu +1)(\mu +2)+3k\mu (\mu +1)(\mu +2)+3{\left(k\mu \right)}^{2}(\mu +2)+{\left(k\mu \right)}^{3}}{{\left(1+k\right)}^{3}\mu^{3}}\right)\\ ={\left(P_{1}+P_{2}\right)}^{3}\cdot \left(\frac{\mu (\mu +1)(\mu +2)+3\mu (\mu +1)(\mu +2)\frac{P_{1}}{P_{2}}+3\mu^{2}(\mu +2){\left(\frac{P_{1}}{P_{2}}\right)}^{2}+\mu^{3}{\left(\frac{P_{1}}{P_{2}}\right)}^{3}}{\mu^{3}\cdot \frac{{\left(P_{1}+P_{2}\right)}^{3}}{P_{2}{}^{3}}}\right)\\ =P_{2}{}^{3}\cdot \frac{\mu (\mu +1)(\mu +2)+3\mu (\mu +1)(\mu +2)\frac{P_{1}}{P_{2}}+3\mu^{2}(\mu +2){\left(\frac{P_{1}}{P_{2}}\right)}^{2}+\mu^{3}{\left(\frac{P_{1}}{P_{2}}\right)}^{3}}{\mu^{3}}\\ =\frac{3\mu +2}{\mu^{2}}P_{2}{}^{3}+3\frac{3\mu +2}{\mu^{2}}P_{1}P_{2}{}^{2}+\frac{6}{\mu }P_{1}{}^{2}P_{2}+{\left(P_{1}+P_{2}\right)}^{3}\\ =\frac{\left(\mu +1)(\mu +2\right)}{\mu^{2}}U_{2}^{3}-\frac{3(\mu +2)}{\mu^{2}}P_{1}^{2}U_{2}+\frac{4}{\mu^{2}}P_{1}^{3} \end{array}$$
